# Quercetin Attenuates the Combined Effects of Zearalenone and Lipopolysaccharide on IPEC-J2 Cell Injury through Activating the Nrf2 Signaling Pathway

**DOI:** 10.3390/toxins15120679

**Published:** 2023-11-30

**Authors:** Chuanqi Wang, Yurong Fu, Ruqi Wang, Qiyuan Wang, Hao Yu, Jing Zhang

**Affiliations:** 1 Jilin Provincial Key Laboratory of Livestock and Poultry Feed and Feeding in the Northeastern Frigid Area, College of Animal Sciences, Jilin University, Changchun 130062, China; wangchuanqi@jlu.edu.cn (C.W.); ruqi21@mails.jlu.edu.cn (R.W.); qiyuanw22@mails.jlu.edu.cn (Q.W.); yu_hao@jlu.edu.cn (H.Y.); 2 Institute of Cereal and Oil Crops, Hebei Academy of Agriculture and Forestry Sciences, Hebei Key Laboratory of Crop Cultivation Physiology and Green Production, Shijiazhuang 050035, China; fuyurong2023@163.com

**Keywords:** quercetin, zearalenone, lipopolysaccharide, oxidative stress, apoptosis, IPEC-J2 cells

## Abstract

Zearalenone (ZEA) is a mycotoxin with an estrogen-like effect that is widely found in feed. Lipopolysaccharides (LPS) derived from Gram-negative bacteria are a common endotoxin, and both toxins have effects on human and livestock health. During animal feeding, ZEA as an exotoxin and LPS as an endotoxin have the potential to co-exist in organisms. At present, other studies have only focused on the hazards of single toxins, but there are fewer studies on the coexistence and interaction between ZEA and LPS. Therefore, a further study to investigate the combined toxic effects of different concentrations of ZEA and LPS is warranted. Quercetin (QUE) is a natural flavonoid compound with strong antioxidant and anti-inflammatory properties. It is unclear whether QUE can mitigate the combined effects of ZEA and LPS. IPEC-J2, isolated from the jejunum of non-breastfed neonatal piglets, is an ideal model for the study of epithelial cell transport, intestinal bacterial interactions, and the nutrient modulation of intestinal function. Therefore, the purpose of the present study was to demonstrate the effect of QUE in alleviating the combined toxic effect of ZEA and LPS on IPEC-J2 cell damage. Cell viability was measured after treating IPEC-J2 cells sequentially with 10, 20, 30, 40, 60, 80, and 100 μM ZEA, 1, 10, 50, and 100 μg/mL LPS, and 20, 40, 60, 80, 100, and 200 μM QUE for 24 h. Based on the cell viability results, 20 μM ZEA and 1 μg/mL LPS were selected as the most suitable concentrations for further analysis. For QUE, 20 μM increased the cell viability, while 40–200 μM QUE decreased the cell viability. Therefore, for the subsequent study, 20 μM QUE was selected in combination with 20 μM ZEA and 1 μg/mL LPS. The results showed that QUE increased the cellular viability and decreased the LDH content more compared to the effects of the ZEA+LPS group. At the gene level, QUE addition up-regulated the expression of Nrf2, HO-1, SOD2, and NQO1 at the gene or protein level compared to those of the ZEA+LPS group. The measurement of tight junction-related genes and proteins showed QUE up-regulated the expression of Claudin, ZO-1, and Occludin genes and proteins more than in the ZEA+LPS group. QUE addition reduced the rate of apoptosis more than that in the ZEA+LPS group. The expressions of Bcl-2 and Bax were examined at the gene level, and QUE addition significantly reduced the Bax gene expression level compared to that of the ZEA+LPS group, but there was no apparent variation in the expression level of Bcl-2. In summary, QUE can alleviate the combined toxic effects of ZEA and LPS on IPEC-J2 cells via modulating the Nrf2 signaling pathway, up-regulating the expression of antioxidative genes, and enhancing the intestinal barrier.

## 1. Introduction

It is well known that the intestinal epithelial barrier, the host immune system, and symbiotic bacteria in the intestinal lumen are dynamically balanced in the gut. The first barrier to encounter natural toxins is the intestinal mucosa. The incidence of colon cancer and common colitis diseases is linked to environmental contaminants, including a common fungal toxin, zearalenone (ZEA) [1,2]. A growing body of evidence has linked mycotoxins to intestinal inflammation [3,4,5]. ZEA, one of the mycotoxins widely present in cereal crops, causes damage to the liver and kidney function of animals and it disrupts intestinal homeostasis and the barrier function. Notably, the intestine is a key physical barrier against ZEA. ZEA is a widely distributed Fusarium toxin that causes reproductive issues and immunotoxicity in animals, and then poses a potential threat to human health through the food chain [6]. Related studies’ proteomic results suggest that ZEA exposure may play a pivotal role in intestinal pathogenesis [7]. In studies where ZEA interfered with the reproductive–immune axis in pigs, it was shown that ZEA-induced toxicity compromised the intestinal barrier by altering the metabolites of intestinal microorganisms (e.g., the reduced production of butyrate and the increased production of LPS) [8]. In studies of the effects of low doses of DON and ZEA on the histological structure and ultrastructure of pigs’ large intestines, it was shown that ZEA may affect the defense mechanisms of the large intestine [9]. Exposure of the gut and its epithelial cell layer to mycotoxins can severely impair intestinal barrier function [10]. Our group has shown that the ZEA treatment of IPEC-J2 cells decrease the cellular viability and increase the content of LDH, and the effect on the cell cycle is characterized by G2-phase stagnation and the inhibition of cell proliferation, thus causing damage to the IPEC-J2 cells [11].

As a common natural dietary polyphenolic flavonoid compound, quercetin (QUE) performs a wide range of pharmacological activities and free radical scavenging abilities, exhibits prominent antioxidant, anti-inflammatory, and antiapoptotic characteristics [12,13,14], and is expected to be a novel feed additive conducive to promoting agricultural development. Studies have shown that QUE significantly inhibits mitochondrial oxidative stress and apoptosis, modulates cellular autophagy, and ameliorates ischemia/reperfusion injury of cardiomyocytes in vitro and in vivo [15,16]. In this study, the QUE mechanism of modulating the cellular toxic action of ZEA and LPS was studied, and in this way, theoretical support that QUE can be a potential feed additive is provided.

## 2. Results

### 2.1. Effect of Quercetin-Mitigated Zearalenone and LPS on the Cellular Viability

**Figure 1:**
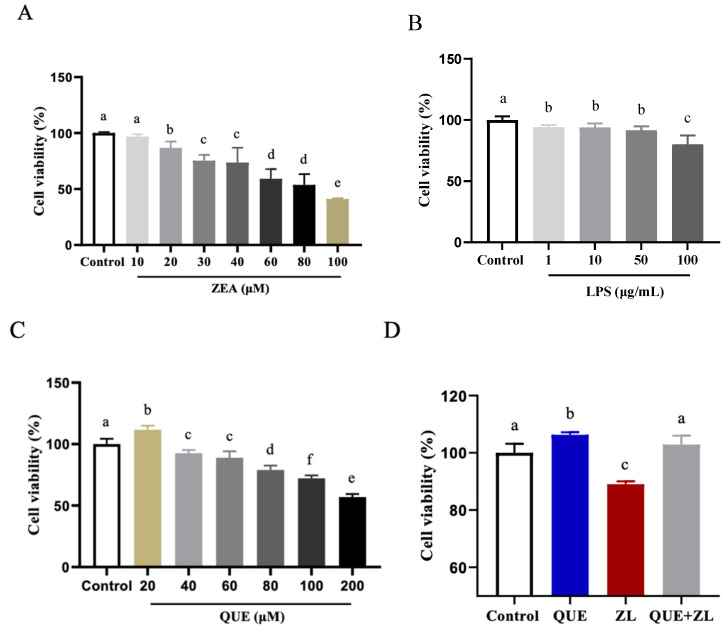
QUE mitigates the effect of ZEA and LPS on the viability of IPEC-J2 cells. (**A**) Cell viability treated with ZEA. (**B**) Cell viability treated with LPS. (**C**) Cell viability treated with QUE. (**D**) Viability of ZEA (20 µM)-, LPS (1 µg/mL)-, and QUE (20 µM)-treated IPEC-J2 cells after 24 h of treatment. The data are presented with mean ± SD (*n* = 6). Different letters indicate *p* < 0.05.

The findings of the present study indicated that cell viability was significantly reduced when treated with ZEA at a concentration of 20 µM alone or LPS at 1 µg/mL alone compared to the control group (*p* < 0.05) (Figure 1A,B). A combination of 20 µM ZEA and 1 µg/mL LPS was chosen for the follow-up study. In addition, different concentrations of QUE (20, 40, 60, 80, 100, and 200 µM) were added to the IPEC-J2 cells, and the cell viability was significantly higher when QUE was treated with 20 µM, but it was significantly lower after 40 µM QUE was applied (as shown in Figure 1C, *p* < 0.05). This further suggests that QUE may have a negative effect on IPEC-J2 cell viability at higher relative concentrations. The combination of 20 µM QUE and ZEA+LPS (ZL for short), shown in Figure 1D, shows that the cell viability was reduced in the ZL group, while up-regulated in the 20 µM QUE group compared to that of the control group. In comparison with the ZL group, the addition of 20 µM QUE resulted in a significant increase in cell viability after the QUE+ZL combination (*p* < 0.05). The IPEC-J2 cells were subsequently exposed to 20 µM QUE, 20 µM ZEA, and 1 µg/mL LPS (the ZEA+LPS group is abbreviated as the ZL group).

### 2.2. Quercetin Alleviates the Effect of Zearalenone and LPS on Cellular Lactate Dehydrogenase

**Figure 2:**
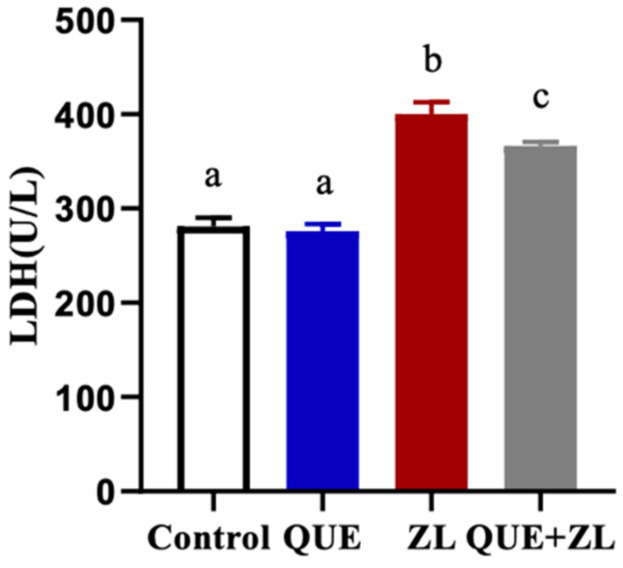
QUE alleviates the effect of ZEA and LPS on lactate dehydrogenase in IPEC-J2 cells. The cells were treated with ZEA (20 µM), LPS (1 µg/mL), and QUE (20 µM) for 24 h, respectively or jointly. The data are presented with mean ± SD (n = 3). Different letters indicate *p* < 0.05.

To further explore the cellular action of QUE, ZEA, and LPS, the IPEC-J2 cells showed no difference in LDH content between the QUE and the control groups, but the LDH content was significantly higher in the ZL group. Additionally, the LDH content was significantly higher in the QUE+ZL co-treatment group (*p* < 0.05). Notably, when compared to the ZL group, the LDH content was significantly lower in the QUE+ZL co-treatment group (as shown in Figure 2, *p* < 0.05).

### 2.3. Effect of Quercetin Mitigation of Zearalenone in Combination with LPS on the Cellular Total Antioxidant Capacity

**Figure 3:**
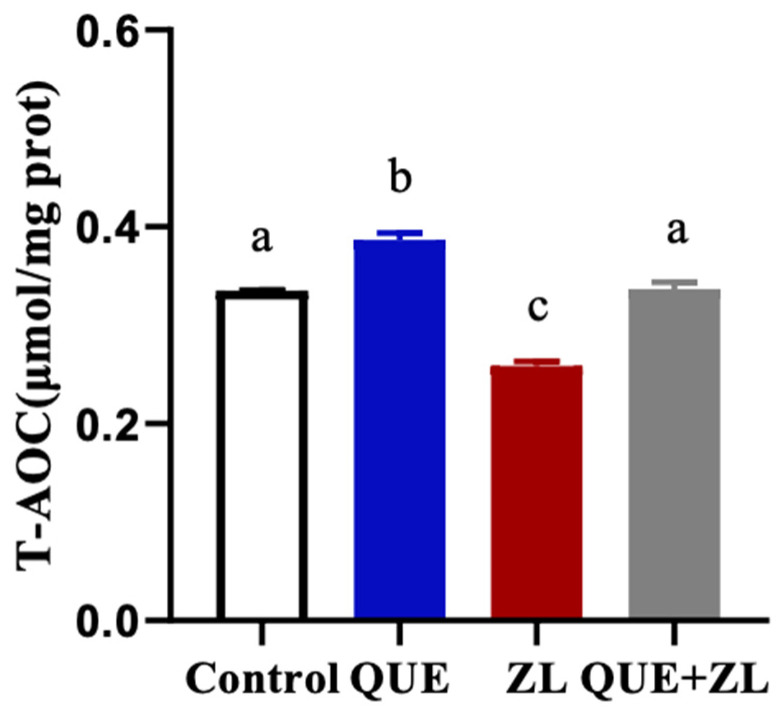
Effect of QUE on the total antioxidant capacity of IPEC-J2 cells treated with ZEA in combination with LPS. The cells were treated with ZEA (20 µM), LPS (1 µg/mL), and QUE (20 µM) for 24 h, respectively or jointly. The data are presented with mean ± SD (n = 3). Different letters indicate *p* < 0.05.

In order to further clarify that QUE has an antagonistic action on the antioxidant activity of ZEA and LPS, an assay was performed. As shown in Figure 3, the T-AOC content was significantly lower in the ZL treatment compared to that of the control group (*p* < 0.05). However, compared with ZL group, the addition of QUE resulted in an elevated T-AOC content after the combined ZEA and LPS combination treatment (*p* < 0.05). 

### 2.4. Quercetin Alleviates the Effect of Zearalenone in Combination with LPS on the Cellular Reactive Oxygen Species

**Figure 4:**
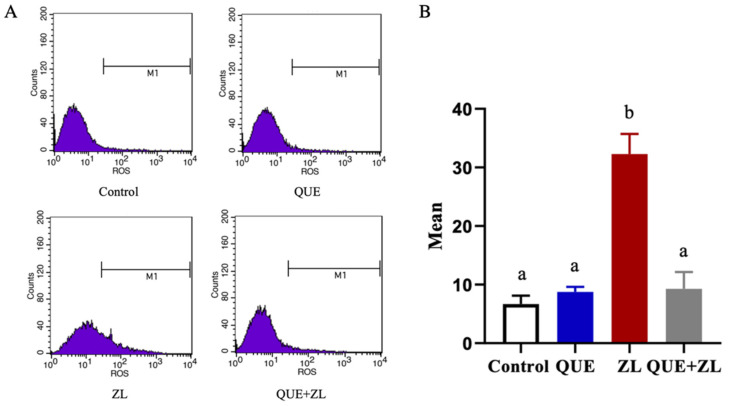
Effect of QUE on reactive oxygen species in IPEC-J2 cells treated with ZEA in combination with LPS. (**A**) After incubation with DCFH-DA, cells were examined via flow cytometry. (**B**) ROS levels in IPEC-J2 cells were analyzed according to MEAN values. The data are presented with mean ± SD (n = 3). Different letters indicate *p* < 0.05.

The mitigating effect of QUE on the IPEC-J2 cells was further clarified. In this study, cellular ROS production was examined, as shown in Figure 4, the ROS level was elevated in the ZL group compared to that of the control group (*p* < 0.05). However, compared with ZL group, the addition of QUE resulted in significantly lower ROS production after the combination treatment (*p* < 0.05).

### 2.5. Quercetin Alleviates the Effect of Zearalenone in Combination with LPS on the Nrf2 Pathway Relative Genes of IPEC-J2 Cells

**Figure 5:**
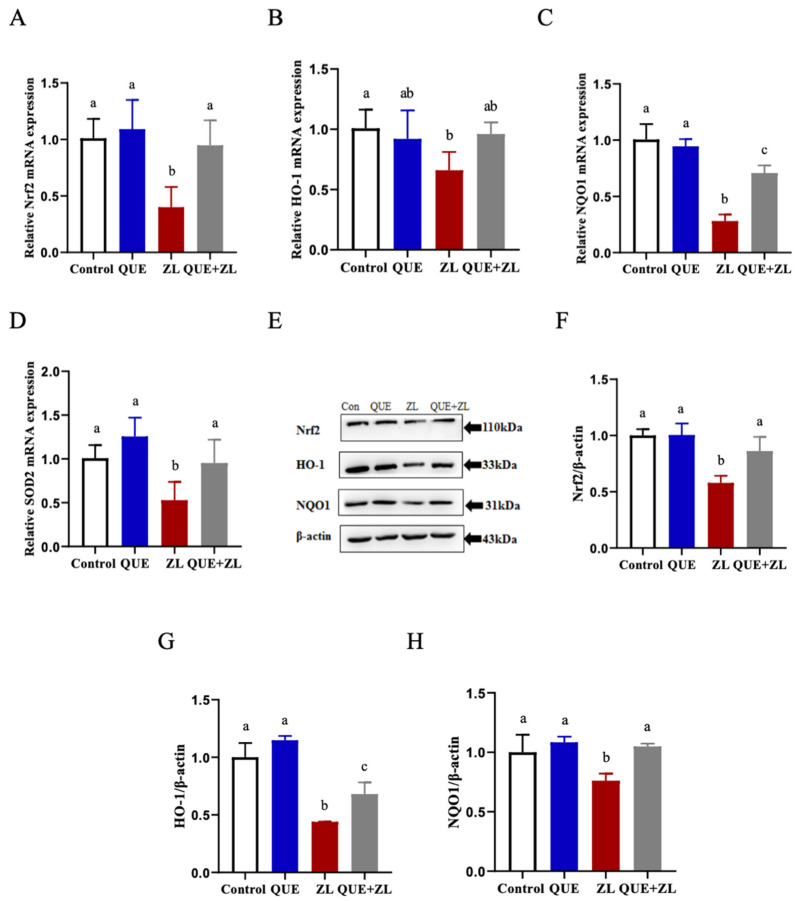
QUE alleviates the effect of ZEA in combination with LPS on the Nrf2 signaling pathway in IPEC-J2 cells. (**A**–**D**) Relative gene levels of Nrf2, HO-1, NQO1, and SOD2. (**E**–**H**) Relative protein levels of Nrf2, HO-1 and NQO1. The data are presented with mean ± SD (n = 3). Different letters indicate *p* < 0.05.

Based on these oxidative stress-related assays and to further clarify the antioxidant capacity of QUE, the genes and proteins related to the Nrf2 signaling pathway were measured in this study (Figure 5). Compared with the control group, the mRNA levels of Nrf2, HO-1, NQO1, and SOD2 were more down-regulated in the ZL group. Compared with the ZL group, the expressions of the genes Nrf2, NQO1, and SOD2 were more up-regulated in the QUE+ZL group (Figure 5A–D, *p* < 0.05). At the protein level, the expression levels of Nrf2, HO-1, and NQO1 proteins were further reduced in the ZL group as compared to the control group. Nrf2, HO-1, and NQO1 protein expression levels were higher in the QUE+ZL group when compared with the ZL group (Figure 5E–H, *p* < 0.05).

### 2.6. Quercetin Alleviates the Effect of Zearalenone in Combination with LPS on Cellular Tight Junctions

**Figure 6:**
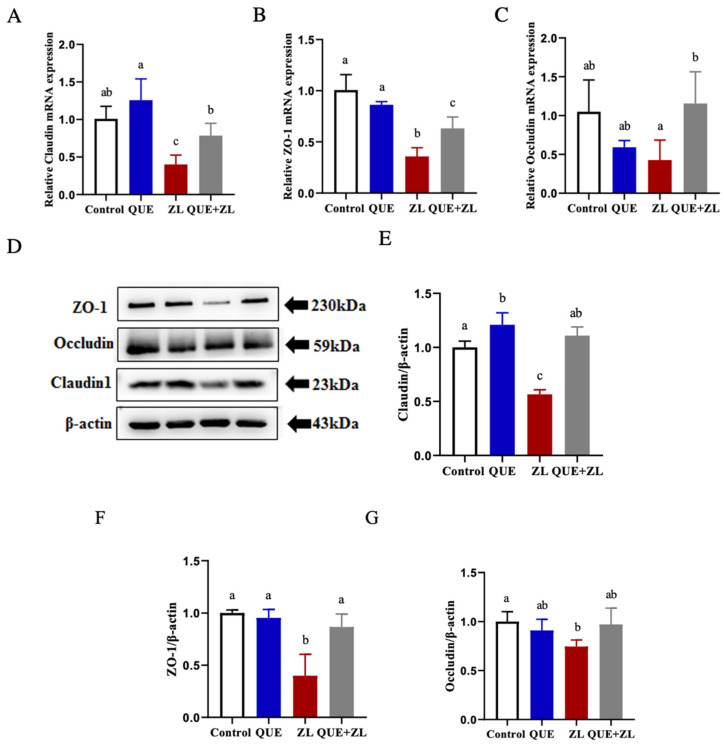
QUE alleviates the effect of the combined action of ZEA and LPS on tight junctions in IPEC-J2 cells. (**A**–**C**) Relative gene levels of Claudin, ZO-1, and Occludin. (**D**–**G**) Relative protein levels of Claudin, ZO-1, and Occludin. The data are presented with mean ± SD (n = 3). Different letters indicate *p* < 0.05.

The regulation of the intestinal barrier is crucial for the preservation of mucosal balance and gut health [17]. Therefore, this study was conducted to investigate whether QUE has an effect on the tight junctions between ZEA and LPS in IPEC-J2 cells. The results showed that at the mRNA level, the expressions of the associated genes, Claudin, ZO-1, and Occludin, were more significantly down-regulated in the ZL group compared to those of the control group (*p* < 0.05). The expressions of Claudin, ZO-1, and Occludin were further up-regulated at the protein level, corresponding to gene expression, compared to those of the ZL group (as shown in Figure 6A–G, *p* < 0.05).

### 2.7. Quercetin Alleviates the Effect of Zearalenone in Combination with LPS on Apoptosis

**Figure 7:**
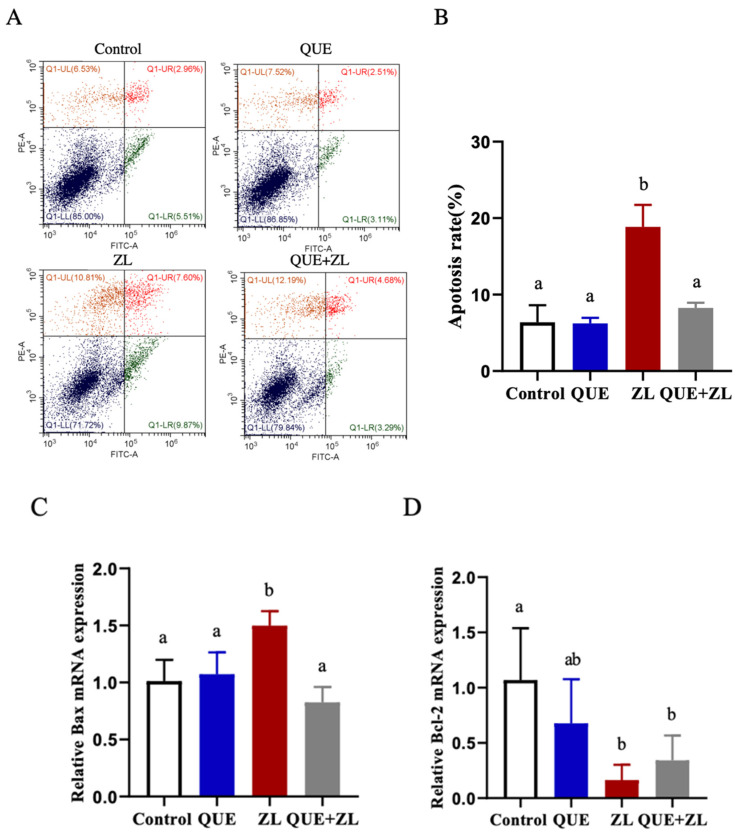
QUE mitigates the effect of combined action of ZEA and LPS on apoptosis of IPEC-J2 cells. (**A**,**B**) The apoptosis rate. (**C**,**D**) The mRNA levels of apoptosis genes. The data are presented with mean ± SD (n = 3). Different letters indicate *p* < 0.05.

Based on the correlation between oxidative stress and apoptosis and to confirm the anti-apoptotic effect, the apoptosis-related indicators were measured in this study. The findings indicated that the apoptosis rate was obviously increased in the ZL group as compared to the control group (*p* < 0.05). Compared with the ZL group, the rate of apoptosis was significantly reduced in the QUE+ZL group (Figure 7A,B, *p* < 0.05). The detection of Bax and Bcl-2 at the gene level showed that Bax was further up-regulated and Bcl-2 was further reduced in the ZL group as compared to those of the control one (as shown in Figure 7C,D, *p* < 0.05). Taken together, the findings indicated that the combination of ZEA and LPS initiated apoptosis, and the supplementation of QUE effectively eased the adverse effects of apoptosis.

### 2.8. Quercetin Mitigates the Effect of Zearalenone in Combination with LPS on Inflammation-Related Genes of IPEC-J2 Cells

**Figure 8:**
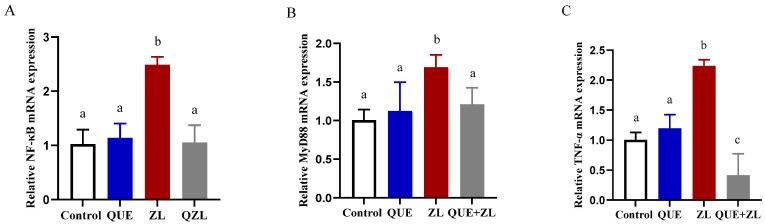
QUE mitigates the effect of combined action of ZEA and LPS on inflammation-related genes of IPEC-J2 cells. (**A**–**C**) The relative gene levels of NF-κB, MyD88, and TNF-α. The data are presented with mean ± SD (n = 3). Different letters indicate *p* < 0.05.

An inflammatory response often occurs in conjunction with oxidative stress. Therefore, in this study, the expression of inflammation-related genes was measured in addition to the oxidative stress detection indexes (Figure 8). The relative expression levels of NF-κB, MyD88, and TNF-α were further up-regulated in the ZL group compared to those of the control group (*p* < 0.05). Meanwhile, compared with the ZL group, the expressions of NF-κB, MyD88, and TNF-α were higher in the QUE+ZL group (*p* < 0.05).

## 3. Discussion

Mycotoxins are often found in livestock as contaminants in farm animal feed [18]. The intestinal tract is an important organ for the digestion and absorption of nutrients, as well as a key barrier against foreign invasive substances and mycotoxins. Mycotoxins enter the body and break down the physical barrier of the animal’s intestinal tract, hindering the rapid response of the body’s immune organs [19,20]. The intestinal epithelial cell layer is the first barrier that prevents mycotoxins from entering lower tissues [21], and multiple functions of the intestinal epithelium contribute to maintain intestinal homeostatic balance [22]. Dysfunctional intestinal barrier integrity caused by toxins and pathogens is associated with a variety of gastrointestinal disorders and diseases [23]. Similarly, pig-related in vivo experiments with deoxynivalenol (DON) and LPS have shown that DON transport is proportional to the concentration and that its presence in feed may have an effect on this through the jejunal mucosa of pig, but LPS does not have that [24]. Therefore, it is important to investigate whether the combined toxic effect of ZEA and LPS can be mitigated using the powerful antioxidant effect of QUE itself and if it is relevant to the abovementioned studies.

The gastrointestinal tract serves as an important defense line against toxins when the accumulated levels of toxins are high, leading to the exposure of toxins to the intestinal epithelium. It is interesting to demonstrate that QUE effectively attenuates the combined effects of ZEA and LPS and remains effective for IPEC-J2 cells. The intestine is the first target of ZEA [25], which affects the integrity and function of the intestinal epithelium and induces intestinal inflammation and oxidative stress [26,27]. In this study, 20 µM ZEA and 1 µg/mL LPS, which caused a significant reduction in cell viability compared to that of the control, were chosen for follow-up testing rather than a greater concentration because, more often than not, in related toxin studies, high concentrations in animals are not affected at low doses [28] nor at non-toxic doses, but contain a greater variety of compound toxins. It is uncertain whether the combined ingestion of toxins could result in a higher risk of adverse health effects than the ingestion of one of the mycotoxins alone could [29,30,31].

Studies showed that ZEA induces intestinal oxidative stress, destroys the structure and function of intestinal microvilli, and hinders the intestinal development of piglets [32]. It has also been found that ZEA and zearalenol (ZEL) do not elicit any intestinal response of pig, which has been attributed to the differences in dietary tolerance between animals at different growth stages [33]. The present study showed that QUE addition reduced the level of cellular ROS production elevated by ZEA+LPS and the changes in LDH. Oxidative stress induced by ROS plays an important role in inflammatory processes, but also plays an important role in organisms for processes such as signal transduction and defense against pathogens [34]. It has been shown that QUE reduced the level of oxidative stress and inflammatory responses in lung epithelial A549 cells; this result that may have been achieved by inhibiting NOX2 production [15]. In INS-1 cells, 1 mg/L LPS reduced the cell viability and insulin secretion, increased ROS, MDA, and superoxide production, while inhibiting SOD activity and FoxO1 nuclear transport [35]. This is a side-by-side validation of the powerful antioxidant properties of QUE. LPS can induce the excessive activation of macrophages (RAW264.7), and activated macrophages induce inflammation and oxidative stress, whereas QUE inhibits LPS-induced macrophage inflammation and oxidative stress [36]. In a related study, a ZEA treatment significantly reduced the expression levels of genes related to the Nrf2/Keap1-ARE signaling pathway, including Nrf2, HO-1, NQO1, SOD1, and GPx1, in IPEC-J2 cells, suggesting that the modulation of Nrf2 pathway can resist endogenous and exogenous oxidative stress in these cells [37,38]. Similarly, this study found that ZEA induced oxidative stress and decreased antioxidant capacity. In Caco-2 cells, it was found that 100 μM QUE could protect the cell barrier function by inhibiting CHOP gene expression and suppressing the Bax/Bcl2 ratio, thereby inhibiting indomethacin- and diclofenac-induced apoptosis [39]. Different reports have also been published regarding in vivo studies in gilts, showing that ZEA alters the expression of key genes and proteins in the Nrf2 signaling pathway of duodenum and can resist ZEA-induced intestinal oxidative stress [40]. In contrast to the present study in an in vitro model, the combined effects of LPS and ZEA, which did not resist oxidative stress, may be simultaneous. The genes and proteins related to the Nrf2 signaling pathway were examined. The results indicated that the combination of ZEA and LPS caused oxidative stress, and the addition of QUE effectively mitigated the effects of oxidative stress. This study demonstrated that the combined toxic action of ZEA and LPS inhibited the antioxidant capacity of IPEC-J2 cells, while the addition of QUE can effectively activate the Nrf2 signaling pathway and increase the antioxidant activity of IPEC-J2 cells, and the activation of Nrf2 pathway can counteract oxidative stress initiated by internal and external factors [37,38]. In this study, ZEA and LPS increased the expressions of NF-κB, MyD88, and TNF-α, while the mRNA levels significantly decreased after the QUE treatment. This result suggests that ZEA and LPS are capable of triggering a combined inflammatory response in IPEC-J2 cells, while the supplementation of QUE reduces the expression levels of inflammation-related genes.

Tight junction proteins, such as Occludin, Claudin, and ZO-1, together form the tight junction complex, and they are commonly used in the response to intestinal tight junction barrier and permeability functions [41]. In this study, the integrity of intestinal tight junctions is closely related to intestinal exposure to toxic and harmful substances, and ZEA and LPS affect intestinal barrier integrity and exacerbate intestinal toxicity. Meanwhile, the expression level of tight junction proteins was significantly higher in the QUE+ZL group compared to that of the ZL group. Similarly to this study, Gu et al. (2021) found that ZEA (20 μg/mL) significantly reduced the expression of tight junction proteins in piglet jejunal epithelial cells [42]. Additionally, ZEA induces DNA breaks, inhibits protein synthesis in Caco-2 cells [43], affects cell viability and cytokine synthesis in porcine intestinal epithelial cells, and induces oxidative stress [26]. Furthermore, ZEA induces local defenses and exacerbates oxidative stress and inflammatory processes in intestinal mucosa of rats, impairing the intestinal barrier [44]. QUE dose-dependently elevated the expression levels of ZO-1 and Claudin1 and activated AhR by enhancing the expression of CYP1A1 in Caco-2 cells [45]. In a study on rat intestinal microvascular endothelial cells, QUE increased the cell migration and expression levels of ZO-1 and Claudin due to LPS-induced intestinal inflammation, cell scorching, and the disruption of the barrier function, while reducing the number of late apoptotic cells [46].

Apoptosis is an autonomous and orderly form of cell death that maintains the homeostatic balance within cells [47]. When apoptosis regulation is imbalanced, excessive apoptosis and cell death can cause severe damage to the body, often leading to the onset of diseases such as autoimmune disorders [48]. In a study assessing the neuroprotective effects of QUE on LPS in adult mice, it was shown that QUE attenuates mitochondrial apoptosis and neuronal degeneration through modulating Bax/Bcl-2 expression and decreasing the activity of cytochrome C and Caspase3 in the cerebral cortex and hippocampus of mice [49]. The antioxidant properties of QUE help prevent ER stress and reduce ZEA-induced apoptosis in human intestinal (HCT116) and renal (HEK293) cells [50]. Similarly, in HCT116 cells, QUE prevented the cytotoxicity of α and β-ZOL, which are derivatives of ZEA [51]. For apoptosis induced by the combination of ZEA and LPS, apoptosis was examined via flow cytometry, and the results showed that the combination of ZEA and LPS caused a significant increase in the rate of apoptosis, which was effectively attenuated by the addition of QUE. Lateral assays of the genes Bax and Bcl-2 at the mRNA level also showed that the addition of QUE down-regulated Bax, but not Bcl-2. Thus, QUE is useful for alleviating the damage caused by the combined effect of ZEA and LPS on IPEC-J2 cells.

The combined effect of different concentrations of ZEA and LPS caused IPEC-J2 and MAC-T cell damage. Regarding the amount of QUE added, the concentrations differed at the cellular level, and there were species-specific reasons for this, but the alleviating effect of QUE was consistent. However, the selection of plant extracts is an effective alternative to traditional physisorption. This study aims to provide theoretical support for QUE as a potential antioxidant feed additive. However, there are still shortcomings in this study, and further in vivo evaluation of the effects of the combined action of ZEA and LPS in animals is needed to achieve the combination of in vivo and in vitro, thus exploring whether the effects of QUE as a potential antioxidant feed additive on oxidative status are specific.

## 4. Conclusions

The combination of 20 µM zearalenone and 1 µg/mL lipopolysaccharide decreased the IPEC-J2 cell viability, increased the lactate dehydrogenase levels, caused reactive oxygen species accumulation, decreased the total cellular antioxidant capacity, and caused oxidative stress. Quercetin inhibits zearalenone–lipopolysaccharide damage to IPEC-J2 cells by regulating the Nrf2 signaling pathway and its downstream genes, up-regulating the expression of antioxidative genes, and enhancing the intestinal barrier.

## 5. Materials and Methods

### 5.1. Chemicals and Reagents

Zearalenone (ZEA, Z2125) and lipopolysaccharide (LPS, SMB00610) were obtained from Sigma (St Louis, Missouri, USA). Quercetin (B20527, purity ≥ 98%) was purchased from Shanghai Yuanye (Shanghai, China). DMEM/F12 (SH30069.03), fetal bovine serum (FBS, SH30406.05), and penicillin-streptomycin (SV30010) were provided by HyClone (Logan, UT, USA). ROS assay kit (S0033S), BCA protein assay kit (P0012), and Annexin V-FITC Apoptosis Detection Kit (C1062S) were purchased from Beyotime Biotechnology (Shanghai, China). Assay kits for superoxide dismutase (SOD, A001-3-2), lactate dehydrogenase (LDH, A020-2-2), total antioxidant capacity (T-AOC, A015-2-1), glutathione (GSH, A006-2-1), and malondialdehyde (MDA, A003-4-1) were purchased from Nanjing Jiancheng (Nanjing, China). The primary antibody against Nrf2 (1:1000, ab137550) as purchased from Abcam (Cambridge, MA, USA). The primary antibodies against HO-1 (1:1000, 10701-1-AP), NQO1 (1:1000, 11451-1-AP), ZO-1 (1:5000, 21773-1-AP), Occludin (1:5000, 27260-1-AP), Claudin 1 (1:1000, 28674-1-AP), β-actin (1:2000, 20536-1-AP), and HRP-conjugated Affinipure Goat AntiRabbit IgG (H+L) (1:4000, SA00001-2) were purchased from Proteintech (Wuhan, China). TRIzol (Cat No. 15596026) and PVDF membranes (Cat No. 24585) were purchased from Thermo Fisher Scientific (Waltham, Massachusetts, USA).

### 5.2. Cell Culture

The cells were removed from the liquid nitrogen freezer for recovery. Cultivation was carried out in 10 cm cell culture dishes by adding a fully configured DMEM/F12 culture medium and setting the temperature of incubator to 37 °C and the concentration of CO_2_ to 5%. The cells were passaged once when they reached about 80% wall confluence.

### 5.3. Cell Viability Assay

The IPEC-J2 cells were inoculated into 96-well plates, and after the cells had reached 60–70% wall confluence, IPEC-J2 cells were separately processed with various concentrations of ZEA, LPS, and QUE. The cells were first treated with 10, 20, 30, 40, 60, 80, and 100 µM ZEA for 24 h, respectively. The IPEC-J2 cells were treated with 1, 10, 50, and 100 µg/mL LPS for 24 h, respectively. The IPEC-J2 cells were treated with QUE (20, 40, 60, 80, 100, and 200 µM) for 24 h. The IPEC-J2 cells were treated with different concentrations of LPS (1, 10, 50, and 100 µg/mL) for 24 h. Cell viability was measured using the CCK-8 kit and recorded with enzyme marker at the absorbance of 450 nm. Based on the above cell viability assay, 20 µM of QUE was screened for subsequent treatment in combination with 20 µM ZEA + 1 µg/mL LPS. The absorbance reading was taken at 450 nm using an enzymatic meter and noted.

### 5.4. Cell Lactate Dehydrogenase Assay (LDH)

The IPEC-J2 cells were inoculated into 6-well plates and treated with ZEA, LPS, and QUE for 24 h after reaching 60–70% growth confluence. After treatment, the cell supernatants were taken for assaying.

### 5.5. Total Antioxidant Capacity (T-AOC) Assay

This involved the same cell processing method as in LDH detection, including the same cell treatment time and dosages of ZEA, LPS, and QUE. After treatment, the cells were collected for further T-AOC analysis with a commercial assay kit. The cells were broken up via ultrasonication, and then centrifuged with the speed of 10,000 rpm for 10 min at the temperature of 4 °C, and the supernatant was removed for assaying. After adding the reagents one by one, the cells were mixed thoroughly, and the absorbance value at 593 nm was measured.

### 5.6. Reactive Oxygen Assay

The IPEC-J2 cells were processed in the same way as they were for the LDH assay. A DCFH-DA probe was configured and prepared in serum-free phenol red-free culture medium at a ratio of 1:1000 on ice. The completed culture was collected, and the cells were collected and suspended in the prepared DCFH-DA and cultured in the incubator with a temperature of 37 °C for 20 min. At the end of the treatment, the cells were washed three times with serum-free cell culture medium. Finally, the ROS was assayed using flow cytometry, and the data were recorded.

### 5.7. Total RNA Extraction and qRT-PCR

The genes expression of Nrf2 pathway and its downstream genes (Nrf2, HO-1, SOD2, and NQO1), two apoptosis genes (Bcl-2 and Bax), three intestinal tight junction-related genes (Claudin-1, ZO-1, and Occludin) three inflammation-related genes (NF-κB, MyD88, and TNF-α), and reference genes (β-actin) in the IPEC-J2 cells were analyzed. The gene primers in the present study were designed with NCBI Primer-Blast and synthesized by Sangon Biotech (Shanghai, China). The total RNA from IPEC-J2 cells was extracted using TRIzol (Thermo Fisher Scientific, USA), and 2000 ng of RNA was used to synthesize the complementary DNA (cDNA) according to the manufacturer’s instructions (TransGenes Biotech, Beijing, China). Briefly, the reaction system included Total RNA, 5 × All-in-One SuperMix, gDNA Remover, and RNase-free water, which was then incubated at 42 °C for 15 min, and the obtained cDNA was used as a template for qRT-PCR according to the instructions of the Green qPCR SupMix kit (TransGenes Biotech, Beijing, China). The reaction system for qRT-PCR consisted of cDNA template, forward primer, reverse primer, 2 × Green qPCR SuperMix, and Nuclease-free water. Then, the cycling condition was 95 °C for 3 min, denaturation for 15 s, and 60 °C for 1 min. The raw qRT-PCR data are expressed as Ct values, and then the 2^−∆∆ct^ method was used to calculate the relative expression levels of the target genes based on previous research [52]. The relative levels of target genes were normalized to the β-actin transcription level in each sample. The differential genes were analysis using Duncan’s multiple comparisons in a one-way ANOVA for statistical analysis. The primers sequence used for fluorescence quantification in this study are shown in Table 1. 

### 5.8. Western Blot

A total of 100 μL of lysate was added to each tube, and after waiting for 10 min in an ice bath, the adherent cells were assembled in an EP tube, and then the protein concentration from collected cells was determined. A protein standard solution of 25 mg/mL was prepared, a standard curve was plotted, the BCA protein working solution was prepared at 50:1, and the absorbance of the sample was measured at 562 nm using an enzyme marker. Then, the protein concentration of the sample was calculated based on the standard curve and the volume of the sample used. The protein was denatured according to the protein concentration (treatment: boiling at 100 °C for 10 min) to a final concentration of 20 μg. The processed protein samples were added to 4% concentrate and 12% separator gel and separated using 80 V concentrate gel and 120 V separator gel. The finished gel is then collected from the electrophoresis tank and transferred to the PVDF membrane (activated in advance with methanol) at 20 V for 24 min using a semi-dry membrane transfer machine. A total of 5% skimmed milk powder was prepared to seal the PVDF membranes at the end of the transfer at 25 °C for 1 h. The primary antibody was subsequently diluted according to the instructions, and after process was completed, the primary antibody was incubated for 4 °C overnight. The next day, the PVDF membranes were washed three times using 1 × TBST for 15 min each. After washing, the secondary antibody was diluted to 1:4000, incubated for 1 h at room temperature, and washed three times with 1 × TBST for 15 min. Finally, a fully automatic chemiluminescence image analysis system was used to scan the PVDF membranes, and the expression of target protein was calculated with ImageJ software (V 1.8.0).

### 5.9. Statistical Analysis

The data were analyzed with SPSS (19.0), using Duncan’s multiple comparisons (Duncan) in one-way ANOVA for statistical analysis. The results are represented as mean ± SD. Different letters show significant differences with *p* < 0.05. Graphs were generated using Graphpad Prism 8.

## Figures and Tables

**Table 1:** Primer sequence list.

Genes	Forward/Reverse Primer (5′-3′)	Accession No.
β-actin	F: TCTGGCACCACACCTTCT R: TGATCTGGGTCATCTTCTCAC	XM_021086047.1
Nrf2	F: AACCAAACCGACAGAAATTGACAAC R: TGGAGAGGATGCTGCTGAAGG	XM_013984303.2
HO-1	F: CCAGGTCCTCAAGAAGATTGCTCAG R: GGGTCATCTCCAGAGTGTTCATTCG	NM_001004027.1
NQO1	F: GTGGAAGCCGCAGACCTTGTG R: CATGGCAGCGTATGTGTAAGCAAAC	NM_001159613.1
SOD2	F: TGTATCCGTCGGCGTCCAAGG F: TCCTGGTTAGAACAAGCGGCAATC	NM_214127.2
Claudin-1	F: TACTTTCCTGCTCCTGTC R: AAGGCGTTAATGTCAATC	NM_001244539.1
ZO-1	F: ACCCACCAAACCCACCAA R: CCATCTCTTGCTGCCAAACTATC	XM_021098856.1
Occludin	F: GCTGGAGGAAGACTGGAT R: ATCCGCAGATCCCTTAAC	NM_001163647.2
Bax	F: CGCTGGACTTCCTTCGAGAT R: CGATCTTGGTGAAGTACTC	XM_003127290.5
Bcl-2	F: GGATAACGGAGGCTGGGATG R: TTATGGCCCAGATAGGCACC	XM_021099593.1
NF-κB	F: CCGTGTCTGCTGCTGCTGATG R: GCCCGCCAAGGAGATGTTGTC	NM_001048232.1
MyD88	F: CTCCATGTCCTCCCTGCCTCTG R: CTCCTCCGCCAGCCCAGTC	NM_001099923.1
TNF-α	F: GCACTGAGAGCATGATCCGAGAC	NM_214022.1
	R: CGACCAGGAGGAAGGAGAAGAGG	

## Data Availability

The results and conclusion presented in this study can be found in the submitted figures and tables. The additional materials and methods supporting this study are available from the corresponding author on reasonable request.
